# Intermittent Hemiplegia in a Boy with Primary Moyamoya Disease: A Case Report from Iran

**Published:** 2017

**Authors:** Reza BIDAKI, Ehsan ZAREPUR

**Affiliations:** 1 Research Center of Addiction and Behavioral Sciences, Shahid Sadoughi University of Medical Sciences, Yazd, Iran; 2 Yazd Diabetes Research Center, Shahid Sadoughi University of Medical Sciences, Yazd, Iran; 3 Student Research Committee, Shahid Sadoughi University of Medical Sciences, Yazd, Iran

**Keywords:** Headache, Seizure, Hemiplegia, Moyamoya Disease

## Abstract

Moyamoya is a rare chronic progressive occlusive cerebrovascular disease. Its manifestation varies from stroke, progressive learning impairment and transient ischemic attack to headache and seizure. There is no accepted medical treatment and surgery usually, is needed. We report here a case of 8 yr old boy referred to psychiatrist outpatient. An eight yr old boy with intermittent hemiplegia was brought to Imam Ali Clinic, Yazd, Iran in 2015 because his headache and medical problem began from 6 yr old. Stress and excitement exacerbated his condition. His first attack was at the age of 6 yr old. During attack, he had incontinence, severe headache, alogia, pallor, claudication and left hemiplegia (Left lower limb). Magnetic resonance angiography (MRA) was done and our diagnosis was moyamoya disease. Moyamoya is a mysterious disease and psychiatrists should consider it in differential diagnosis of alogia and plegia.

Acute management of this disease is mainly symptomatic. Nowadays, surgery is a good choice and early diagnosis of this disease can change our patient’s life.

## Introduction

Moyamoya disease (MMD) is multifocal occlusive arterial disease (distal), hereditary and linked to chromosome 17 (1). Moyamoya is a Japanese expression for something hazy like cigarette smoke and its incidence is higher in Japan (2). MMD occurs mostly in children in Asia. It is a rare cerebrovascular disease. The annual rate of newly diagnosed cases of MMD in 1994 was 0.35 per 100000 associated with 6% of childhood acute strokes. Spontaneous occlusion of circle of Willis usually happens in this disease (3, 4). In China the prevalence is 3.92/100 000 (5). 

Although often reports about MMD are related eastern countries like Japan, but it is probably in other countries and we intend to report a case of 8 yr old boy referred to our clinic.

## Case report

An Iranian 8 yr old boy was brought to Imam Ali Clinic, Yazd, Iran in 2015. The low socio economic area in north of Yazd this clinic is a special clinic related to Shahid Sadoughi university of medical sciences, Yazd, Iran because of intermittent hemiplegia and headache. His medical problem began from 6 yr old. As mother’s history, stress and excitement exacerbate his condition. His past medical history was clear except a head trauma following falling at the age of 2 yr old and he had no hemorrhagic events, loss of consciousness or fracture after head truma. His birth weight was 3140 gr and now was 19 kg. He was lower than normal limit in spite of normal diet, probably due to disease. Her parents had not consanguinity of marriage. 

He had no congenital anomaly, language problem or developmental delay or learning disorder. Informed Consent was taken from his mother. 

IQ test was normal and he was a good student without academic problem. His mother did not mention childbirth complication but she experienced noticeable stress during pregnancy.

The first attack was started in 6 yr-old following an emotional condition. Nearly every two weeks, he experienced an episode. During attack he had incontinence, severe headache, alogia, pallor, claudication and left hemiplegia (Left lower limb). He had not vomited and visual disturbances. The child had so much stress about his attack and was conscious on his disease.

**Paraclinical Findings**

**Magnetic Resonance angiography findings:**

**Fig 1:**
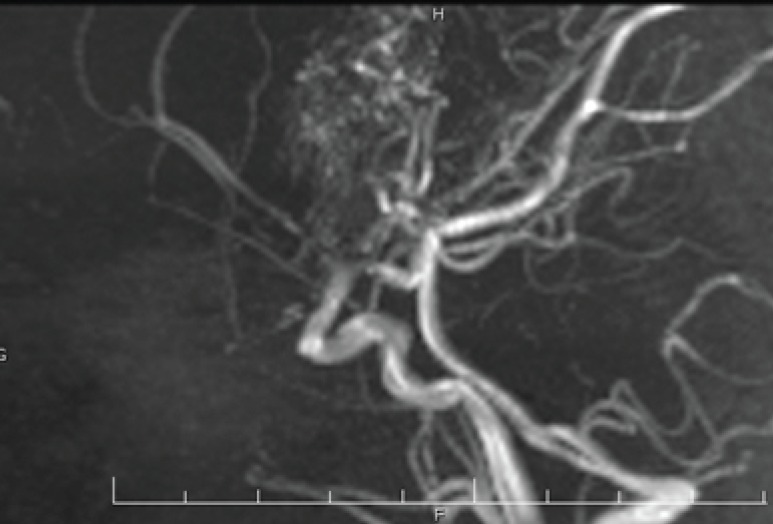
Stenosis of supraclinoid internal carotid vasculature with a proliferation of collateral vessels (Puffs of smoke appearance

**Fig 2:**
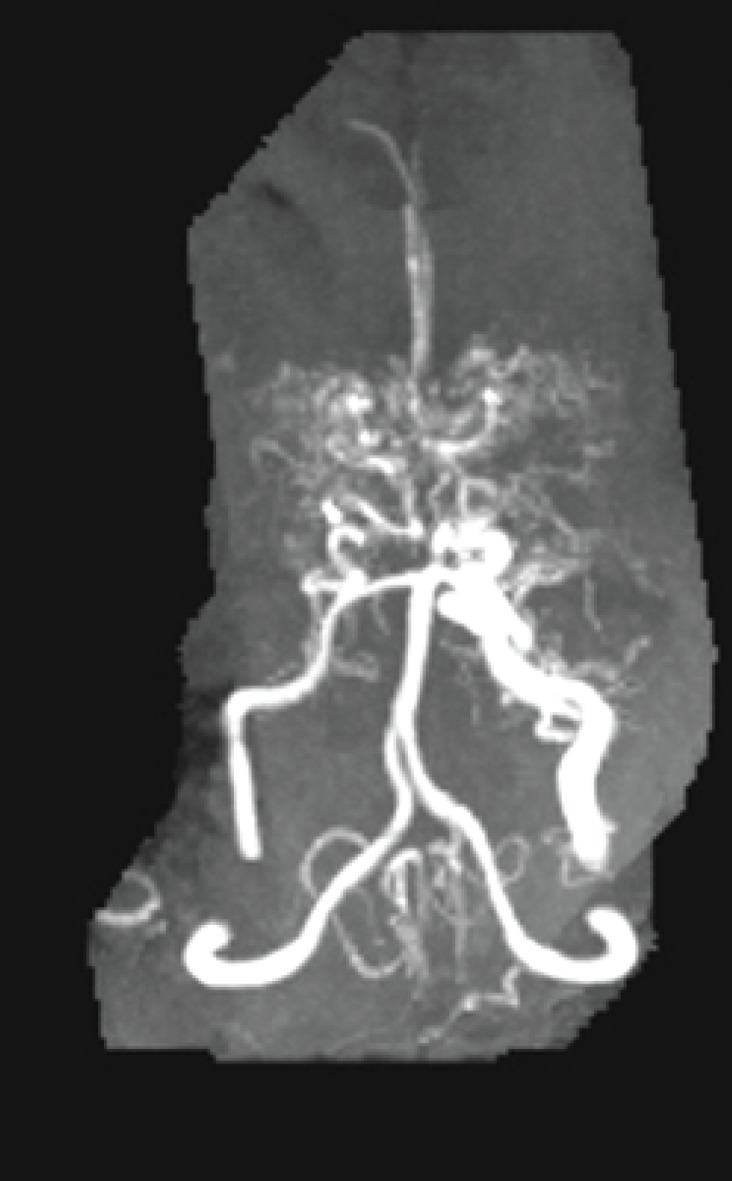
Stenosis of supraclinoid internal carotid vasculature with a proliferation of collateral vessels (Puffs of smoke appearance

The marked stenosis of supraclinoid internal carotid vasculature with a proliferation of collateral vessels (Puffs of smoke appearance) suggestive MMD were noted (Figure 1, 2).

His response to tablet sodium valproate (200 mg daily) was better than nortriptyline 25 mg at night and lamotrigine 25 mg/daily.

**Follow-up**

We visited him every 2 months. Following prescription of sodium valproate, he had one attack only, although we suggested for consultation with a neurosurgeon.

## Discussion

MMD is a chronic cerebrovascular disease and often presents with hemorrhage or transient ischemic attack, leading to cerebral stroke.

The most common differential diagnosis of this condition is seizures and strokes. Some diseases have relative association with this disease (Patients with structural congenital heart disease). Conversion disorder is the other differential diagnosis (6). The investigations were negative for conversion disorder and malingering and he had no primary or secondary gain or attention sicking.

Age is one of the most important factors. Symptomatic presentation of MMD depends on it and is related to prognosis. Nearly half of the patients observed from 1961 to 1980 were children below 15 yr (7). The symptoms were varied from no symptoms to transient or even fixed disease but transient ischemic attack was predominated in children. Adults usually present with intracranial hemorrhage (ICH), and this can lead to paralysis and severe headache.

TIA can manifest as episodes of hemiparesis, speech and visual disturbance, sensory impairment and we must consider MMD especially when the symptoms are related to emotional states, cry or physical activity (8). 

Yamauchi et al. reported familial occurrence of MMD as 5%-15% (9). Actually, mother-to-child inheritance was observed in a few cases (about 5 cases of 68 patients) and father-to-child inheritance was so rare (9). Our case had no positive familial history.

The diagnosis of MMD is confirmed by radiographic studies such as CT scan and MRI as well as MRI angiography. The characteristic findings are arterial narrowing (puff of smoke, a commonly low-density area in the white matter of the temporal lobe) and clear collaterals. The workup begins with brain CT scan and we can see hypodensity small areas but MRA should be performed for the clear-cut diagnosis, follow-up and detect of acute infarcts regions (10, 11). Because of the noninvasive nature of MRI, MRA is primary diagnostic imaging modality for MMD (12). Earlier diagnosis may prevent additional neurologic deficits.

Currently, there is no accepted medical treatment for reversing of this progressive disease and management mainly is symptomatic. Some patients stabilize clinically without intervention, but this condition usually occurs after they have experienced neurologic disability. However, there are some evidence for the use of anticoagulants/antiplatelet agents and vasodilators. 

Aspirin is a suitable choice (80 mg daily those younger than 6 yr). The calcium channel blockers are the other medication class. These drugs may be effective in reducing symptoms (like headache) or in reducing both the frequency and severity of refractory TIA (11).

The literature supports of revascularization procedures through surgery and surgery can reduce cerebral ischemic events especially in patients with recurrent or progressive course Non-surgically treated MMD prognosis is not clear yet (13).

In this case, patient responds to sodium valproate. It can cause thrombocytopenia and can help blood circulation.

Besides, it can help to relieve tension and anxiety and then reduce vasoconstriction (14).

As we know, MMD has a progressive nature. We do surgical treatment to prevent ischemia or infarction. 

In addition, antiplatelet treatment has used in treating in MMD but we do not have enough evidence base medicine in this case (15).

**In Conclusion, **the physician should know Moyamoya and its neurologic presentations in childhood. Physicians should consider it in differential diagnosis of seizure, headache, plus plegia and treat resistant cases to medical treatment in children.
